# Interprofessional approach to fall prevention in hospital care

**DOI:** 10.1590/1980-220X-REEUSP-2023-0239en

**Published:** 2024-07-05

**Authors:** Anna Carolina da Silva Albertini, Marina Peduzzi

**Affiliations:** 1 Universidade de São Paulo, Escola de Enfermagem, São Paulo, SP, Brazil.; 2 Hospital Sírio-Libanês, São Paulo, SP, Brazil.

**Keywords:** Patient Care Team, Interprofessional Relations, Accidental Falls, Hospitals, Grupo de Atención al Paciente, Relaciones Interprofesionales, Accidentes por Caídas, Hospitales

## Abstract

**Objective:**

To understand the perception and experience of health professionals regarding fall prevention practices in hospital inpatient units.

**Method:**

This is a qualitative exploratory and descriptive case study based on the Canadian framework of interprofessional competences. Data was collected from two focus groups, with different health professionals in each group, and thematic content analysis was used.

**Results:**

Five categories were drawn up which showed intense convergence between the participants of the two focus groups, within the context of fall prevention practices: communication between professionals and patients/carers for fall prevention, interprofessional communication for fall prevention, clarification of roles for fall prevention, health education about risk and fall prevention and continuing education for fall prevention.

**Conclusion:**

Teamwork and collaborative practice are important for achieving good results in the prevention of falls in hospital care, but for this to happen, health professionals need to acquire the necessary competences for collaborative action.

## RESUMEN

**Objetivo:**

Conocer la percepción y experiencia de los profesionales de la salud en relación a las prácticas de prevención de caídas en unidades de internación hospitalaria.

**Método:**

Se trata de un estudio de caso cualitativo, exploratorio y descriptivo, basado en el marco canadiense de competencias interprofesionales. Se recogieron datos de dos grupos focales, con diferentes profesionales sanitarios en cada grupo, y se utilizó el análisis de contenido temático.

**Resultados:**

Se elaboraron cinco categorías que mostraron una intensa convergencia entre los participantes de los dos grupos focales, en el contexto de las prácticas de prevención de caídas: comunicación entre profesionales y pacientes/cuidadores para la prevención de caídas, comunicación interprofesional para la prevención de caídas, clarificación de roles para la prevención de caídas, educación sanitaria sobre riesgos y prevención de caídas y formación continuada para la prevención de caídas.

**Conclusión:**

El trabajo en equipo y la práctica colaborativa son importantes para conseguir buenos resultados en la prevención de caídas en la atención hospitalaria, pero para ello es necesario que los profesionales sanitarios adquieran las competencias necesarias para la acción colaborativa.

## INTRODUCTION

Falls within the hospital environment are a key cause of concern for healthcare professionals, as approximately 37.3 million falls require care each year provoking around 684,000 fatalities^(1)^.

The occurrence of this adverse event in the hospital environment implies an increase in morbidity and mortality, longer hospital stays, physical and psychological damage, with a consequent rise in healthcare costs and increased judicialization of health services^(2-4)^.

Falls are the third most reported adverse event in Brazil notified by the ANVISA (National Health Surveillance Agency) Notivisa system^(2)^. Damage occurs in 30% to 50% of reported cases, including mild damage (e.g. abrasions, hematomas), moderate damage (bleeding, sprains), severe damage (femur and hip fractures and skull trauma) and catastrophic damage (partial or total loss of function and death)^(1-4)^.

There are several factors that contribute to an increase in the number of falls in hospitalized patients: balance disorders related to loss of lower limb muscle strength and altered gait, hypotension, anemia, paresis, osteoarthritis, neurological disorders, amputation, cachexia or severe obesity, sensory impairment (sight, hearing, touch), fasting, severe pain, dressings that can impair the patient’s mobility and the use of walking devices (crutches, walking sticks, walkers)^(1-5)^.

There are many recommendations for preventing falls, among the most common being the importance of training healthcare professionals and guidance for patients, family members and carers on good practices for preventing falls^(6-12)^. The guidelines refer to the need to keep the bed in a low position with the railings raised and locked, the alarm button always within reach so that the care team can be called when necessary, personal items also within reach of the patient and to maintain visual signage with identification according to the patient’s risk of falling (wristband or bedside signage), in order to alert the entire care team involved in the care^(6-12)^.

Periodic rounds of the patient’s room are also a strategic practice for minimizing the risk of falls, as the care team is able to ascertain the patient’s needs in advance (going to the bathroom, putting on glasses, prostheses, etc.), before the patient gets up or moves, especially if the patient is unaccompanied, a condition that further increases the risk of falls^(6-12)^.

The carers play an essential role in the context of fall prevention, as they are the first to alert and activate the care team at times of greater risk and danger to the patient. The empowerment of the patient and family members/carers, with the involvement of everyone in the care, contributes to positive results for falls prevention, since this practice is related to patient-centred care and, consequently, to interprofessional practice^(6–12)^.

The literature points to the need for the whole healthcare team to be involved in preventing falls in hospital care, so that all professional categories involved in direct patient care should be well trained to correctly identify patients at moderate and high risk of falling and be able to propose and implement effective preventive strategies, with the active participation of the patient and their family/carer^(6–12)^.

The aim of this study was to analyze the perception and experience of health professionals in the multiprofessional team regarding fall prevention practices in a hospital inpatient unit.

## METHOD

### Type of Study

This is a qualitative, exploratory and descriptive case study. This provides an in-depth investigation of a phenomenon in the place where it occurs, and is used to approach a set of contemporary events that have not yet been explored^(13)^.

The aim of an exploratory study is to analyze a scarcely studied topic, as well as unknown or new phenomena, research new problems, identify concepts and establish priorities for new studies to be carried out. Descriptive research seeks to observe, record and specify the characteristics of the phenomenon to be analyzed and to accurately show the dimensions of a context or situation. The researcher must define what will be observed and what data will be collected^(13)^.

The case study provides an in-depth investigation of a phenomenon, in the place where it occurs, and is used when the aim is to investigate the how and why of a set of contemporary events that have not yet been explored^(14)^.

### Study Site

The study was carried out in a hospital located in the city of São Paulo, which caters for more than 40 specialties, with 512 inpatient beds, an Emergency Department, a Surgical Centre, Critical Units and Advanced Medicine Centers. The hospital has been accredited by the Joint Commission International (JCI) since 2007.

### Study Participants

Health professionals from different areas took part in the study, with both technical and higher education qualifications, who met the three inclusion criteria: they had been working in the hospital studied for at least a year, had experience of working in an inpatient unit and, in particular, with fall prevention care, and ensured the participation of the different professional categories.

### Data Collection Procedures

A focus group was chosen as the data collection method, since the interactions between the participants could contribute to producing and analyzing the perceptions and experiences of the different professionals in the healthcare team about fall prevention care. The study’s main researcher acted as coordinator in both focus groups, facilitating the interactions through guiding questions that dealt with the Flow and Guidelines for Falls Prevention applied in the hospital studied, the participants’ experiences regarding the actions and responsibilities of each professional category and the participation of other professionals in falls prevention practices, and the perceptions and experiences regarding the participation of patients and their families/carers.

Two focus groups were held to which two different professionals from the following areas were invited: nursing (nurse and nursing technicians), pharmacy, physiotherapy, medicine, nutrition, psychology and social work. Five health professionals took part in Focus Group 1 (FG1): a pharmacist, a nurse, a doctor, a nutritionist and a nursing technician. The social worker, physiotherapist and psychologist did not take part due to the high demand for care and the need for support from these professionals in the Covid-19 scenario on the date when the focus group had been scheduled by mutual agreement with the participants. Eight health professionals took part in Focus Group 2 (FG2): a social worker, a nurse, a pharmacist, a physiotherapist, a doctor, a nutritionist, a psychologist and a nursing technician.

Each of the focus groups (1 and 2) also included the participation of an observer: both were nurses with master’s degrees in health sciences and had been working in the hospital studied for 19 years and 14 years respectively.

Although data collection took place during the Covid-19 pandemic, it was possible to hold the two focus groups in person, in a meeting room with image and voice recording facilities, after the participants had given their consent by signing the Informed Consent Form. The focus group began with a brief introduction of the participants, an explanation of the purpose of the research and a presentation of the institution’s falls prevention protocol. The meetings took place in June 2021, each lasting 2 hours.

The guiding questions for the focus groups were as follows:

1. What is your opinion of the Flow and Guidelines for Falls Prevention currently applied in the institution? Is there any practice, any aspect that deserves to be better discussed?
2. How can each of you, thinking about the role you play in your professional responsibilities and competences, contribute to Fall Prevention in the Hospital Inpatient Unit?
3. We want to hear about your experiences of involving patients and their families/carers in fall prevention practices.
4. We want to hear about your experiences of the participation of professionals from different areas in fall prevention practices.
5. In your opinion, what are the main challenges and difficulties for Falls Prevention in the hospital inpatient unit?
6. To close, would anyone like to make any further suggestions, contributions or comments?

### Data Analysis

In order to analyze the data generated from the focus group, the study used Bardin’s content analysis method^(15)^, which seeks to know what is behind the words, therefore latent, seeking to get closer to the reality studied through the reports and exchanges between the participants and the context of each report, i.e. the professional category of the participant. The three stages of thematic content analysis were followed: pre-analysis, with transcription of the two focus groups by the main researcher and floating reading of the empirical material; exploration of the material, with coding and, based on this, the construction of subcategories and categories with repeated revisions going back to the reading of the empirical material and validation with the second researcher, the study supervisor; and treatment of the results obtained and interpretation, in which inferences and interpretations were proposed for the construction of categories^(15,16)^.

Each group was examined separately and then the two groups were cross-analyzed. At the start of the analysis, deductive logic was used, based on the Canadian model of collaborative competences, but gradually inductive logic was also used to develop subcategories and categories. The entire content analysis process was based on the theoretical framework of teamwork and interprofessional collaboration^(17-22)^ and collaborative competences^(23-26)^, and also supported by the literature on fall prevention.

### Ethical Aspects

The study observed ethical and legal aspects contained in Resolution 466/2012 of the National Health Council (CNS)^(27)^, and it was submitted for evaluation to the Research Ethics Committee (CEP) of the hospital studied, with approval (opinion n°4.210.393, of 2020 and opinion n°1628, of 2020). The research was carried out after the participants of the two focus groups had signed the Free and Informed Consent Form. In order to guarantee the confidentiality and protection of the participants’ data, we identified the participants by the code for Focus Groups 1 and 2 and by professional category: social worker (AS1 and AS2), nurse (EN1 and EN2), pharmacist (FA1 and FA2), physiotherapist (FT1 and FT2), doctor (ME1 and ME2), nutritionist (NU1 and NU2), psychologist (PS1 and PS2), nursing technician (TE1 and TE2).

## RESULTS

Five health professionals took part in Focus Group 1 (FG1) at the hospital where the research was carried out: a nurse (EN1), 32 years old, nine years of training and eight years of work; a pharmacist (FA1), 33 years old, 12 years of training and two years of work; a doctor (ME1), 63 years old, 40 years of training and 38 years of work; a nutritionist (NU1), 28 years old, seven years of training and two years of work; a nursing technician (TE1), 42 years old, nine years of training and seven years of work.

Eight health professionals from the hospital where the research was carried out took part in Focus Group 2: a social worker (AS2), 35 years old, 13 years of training and 11 years of work; a nurse (EN2), 30 years old, eight years of training and seven years of work; a pharmacist (FA2), 32 years old, eight years of training and two years of work; a physiotherapist (FT2), 37 years old, 13 years of training and 11 years of work; a doctor (ME2), 41 years old, 16 years of training and nine years of experience; a nutritionist (NU2), 44 years old, 18 years of training and two years of experience; a psychologist (PS2), 57 years old, 34 years of training and seven years of experience; a nursing technician (TE2), 54 years old, 23 years of training and 21 years of experience.

In both Focus Groups, all the participants felt comfortable interacting and expressing their experiences and perceptions.

**Figure 1:**
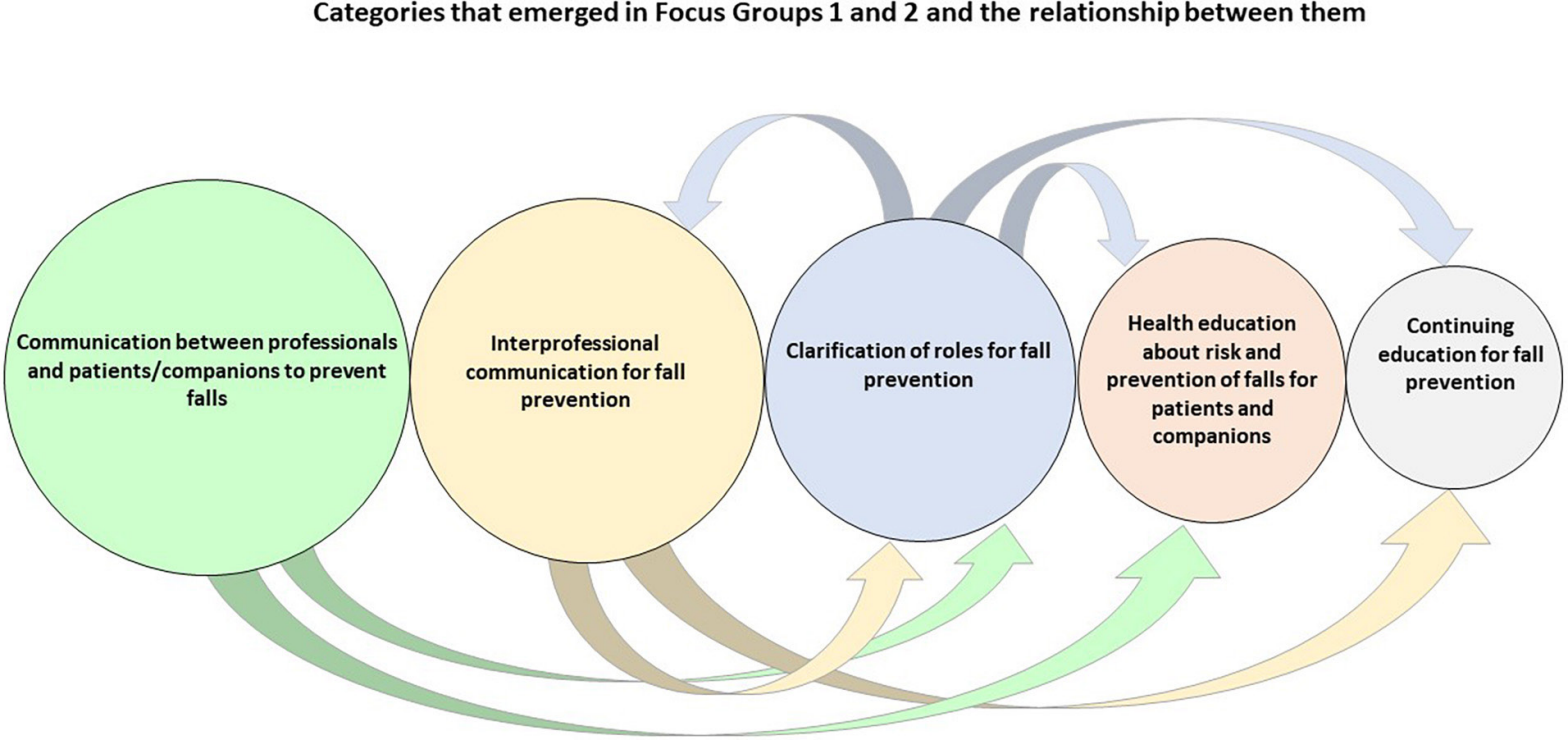
Categories constructed from the analysis of the empirical material and the relationship between them signaled by arrows.

The analysis of the empirical material from FG1 and FG2 resulted in the creation of five categories that showed intense convergence between the participants in the two focus groups, although divergences were also analyzed between the participants in each focus group and between the two groups (FG1 and FG2), as shown in Figure 1.

### Communication Between Professionals and Patients/Carers for Fall Prevention

The participants in the two focus groups (FG1 and FG2) showed that in their perception and experience, communication between healthcare professionals and patients/carers is a key element in fall prevention practices. Communication between professionals and patients/carers was the predominant theme in both focus groups, and was seen as effective in prevention actions if accompanied by active listening, in the sense of showing the patient a genuine interest in hearing them out, empathy and the use of easy-to-understand language. Health professionals also pointed out that communicating with patients/carers about risk factors and fall prevention measures requires identifying the patient’s needs and preferences in the context of care. In other words, interaction and communication around fall prevention needs to make sense to patients and carers at the moment it occurs.

“*How many times do we go in and talk to the patient and they don’t look at us* (health professionals)*? He’s fiddling with his mobile phone, he doesn’t want to* (...), *he’s saying to you: ‘I don’t want to talk to you’, so you* (the healthcare professional) *have to have the sensitivity to come back and say ‘I’ll come back to this patient later’*” (NU2).

“*As a nursing technician, I believe that the contribution I could make to the patient would be active listening, because it’s quite different to listen than to hear. Listening refers to the senses and sometimes we say to the patient: ‘Here’s the folder, call me when you need to, the bars will have to stay up and you can sign here’* (referring to the awareness document on fall prevention measures). *Then he* (the patient) *signs it, what percentage of the content has this patient actually absorbed*? (TE1).

The participants in FG2 mentioned that in their experience, establishing a bond between patients and health professionals is also related to good communication between them and helps to ensure patient safety, as the bond facilitates greater adherence by patients and carers to the preventive recommendations proposed by the professionals. However, the relationship between bonding and effective communication in the prevention of falls did not appear in FG1.

“*So when you* (health professionals) *already have a bond* (with the patient) *and when you already have this connection, this experience of adherence is 10 million times more effective, efficient* (...). *And when a patient is admitted on the same floor, the nurse says: oh, doctor, we* (the healthcare team) *already know the patient who is being admitted to that unit and we already know their particularities. So I think that the presence of a bond prior to hospitalization improves adherence and* (the patient’s) *experience of our care practices as a whole, including falls prevention*” (ME2).

Most of the participants mentioned communication with patients and carers as a component that contributes to patient safety. However, one of the professionals referred only to the involvement of the carer in patient safety care, referring to the need for their understanding of the risk factors for falls.

“*The carer is in the room for most of the time, so when a medication is taken and there is an adverse reaction, agitating the patient and the carer doesn’t share it* (with the team), *but it’s important to have the information* (medication effects) *and share what happens in the room to prevent falls*” (FA1).

There was a divergence between groups FG1 and FG2 regarding the interference of technology in falls prevention care, since FG1 pointed out the importance of technology, but FG2 asked how much technology can help prevent falls with bed alarms.

“*And I believe that the prevention, prevention guidelines* (for falls) *that we* (the healthcare team) *use, have improved a lot in relation to, for example, the institution of beds with alarms in the hospital* (beds with movement alarms that warn the team when the patient tries to get up on their own, without asking for help), *especially for patients who are in critical units, that it does happen that they* (patients) *get up, try to get up and this has helped us* (the healthcare team) *a lot, to alarm the bed and for us* (the healthcare team) *to enter the room”* (EN1).

“*The hospital beds, I think they’re state-of-the-art, but in my opinion they’re not functional for the risk of falls, because you* (the healthcare professional) *enter the room, you orientate, you lift the* (bed) *rails, and when you arrive, the patient is in the bathroom! The* (bed) *rails are up, so where did he* (the patient) *get out? Through the edge* (of the bed)*... Even with the alarm on... Because there’s a big space where he goes through* (space without rails at the foot of the bed). *So often the patient gets out, goes back to bed and you* (the healthcare professional) *don’t even know that the patient got out*” (TE2).

### Interprofessional Communication to Prevent Falls

In both focus groups, health professionals’ perceptions and experiences of the need for interprofessional communication for the best performance of care-related actions to prevent falls were presented.

“*Exchanging information is very important, passing on what happened last shift, the patient is not a fixed* thing (...). *He’s volatile during the day, he changes a lot depending on the medication the doctor has given him, which is often necessary* (...), *so there’s a variation in muscle strength, level of consciousness* (...). *In the morning, he was on one mood, in the afternoon he’s another* (...), *this exchange of information between shifts is very important*” (FT2).

One FG1 participant mentioned a learning experience at work that resulted from interprofessional communication with an exchange of knowledge and collaboration, which enabled better performance in actions related to fall prevention care.

“*I have a great experience working with physiotherapists* (...), *I think it’s exceptional because it’s a connection that we* (nursing technicians and physiotherapists) have (...). *I’m there doing a procedure; the physiotherapist comes in* (...). *I’m changing a nappy, and the physiotherapist says to me: “Do you want a bath now? I’m going to help you because this patient, from what I worked on yesterday, I know he has a difficulty here or there. Sometimes I* (the nursing technician) *have already received the patient’s shift, I already know what their difficulties are, but I haven’t really felt what that difficulty will be like, so I think it’s very good to have the synergy we have there*” (TE1).

The participants in Focus Group 2 mentioned that there is difficulty in interprofessional communication for physical and technological reasons, relating this difficulty to the use of two systems in the electronic medical record, one of which is used by the medical team and the other by the other professional categories, which makes it difficult to access the information recorded due to the time taken to use both systems, which reduces the availability of the professional at the bedside and the possibility of communication and interaction with patients/carers and other health professionals.

“*I don’t have access to what the pharmacist writes, what the nutritionist writes, what the physiotherapist writes, what the nursing technician writes, what the psychologist writes... I have to access the system* (electronic medical record system) *and even then, it should be automatic. I can access what you’re writing, it should be much easier, because without a shadow of a doubt my action depends a lot on the information I have there, that I see. What we always hear is “but you just have to access the system”, “the system is slow, the system takes too long, the system doesn’t know what...” Then the second system* (another electronic system for making prescriptions and viewing patient records) *takes 1 hour to release the prescription, and then you’ve spent 15 minutes just in theory and practice, there in the exchange where you could be spending those 15 minutes encouraging patient autonomy and stimulating the family, you’re trying to get the system right to find out what the pharmacist or the nutritionist thought, because you can’t...*” (ME2).

### Role Clarification for Falls Prevention

Role clarification also converged in the two Focus Groups as an element that contributes to falls prevention and expresses the understanding that health professionals have of the need to recognize the actions and knowledge of their own professional practice, as well as the actions and knowledge of the other areas that make up the team and are involved in falls prevention care. The process of clarifying the roles of one’s own professional area and those of the others that make up the team leads to the possibility of interprofessional co-responsibility in fall prevention actions, which is one of the characteristics of integrated teamwork with interprofessional collaboration^(23-26)^.

However, the results show that the health professionals who took part in the study are particularly aware of their specific contribution from their health profession and less aware of the actions and responsibilities of the other team members.

“*As a pharmacist, the experience I’ve had... I think I’ve had more experience now because I’m part of the audit team* (internal audit related to the falls prevention bundle) *and when we go in to do this audit, they see that we as pharmacists are also caring, because of some guidance that the nursing team has given. ... that other professionals are also keeping an eye on the risk of falls and when we do the pharmaceutical interview, we also advise on the adverse reactions of the medicines and often they don’t realize that they are using the medicine that has a risk of falls*” (FA1).

The participants in both Focus Groups mentioned that falls prevention could be the responsibility of all members of the healthcare team. However, it was also evident in FG1 and FG2 that in the participants’ perception, the practices carried out in the hospital environment to ensure falls prevention are mainly the responsibility of the nursing team.

“*I see that a lot ends up being in the hands of nurses* (...), *a lot of guidance* (...) *they have a lot of responsibility for this part of comprehensive care and sometimes this could be multiplied, divided between the other health professionals. In fact, the whole team could give advice on fall prevention, after all, it’s patient care. For example, we have up to 24 hours to do our triage and then we often end up going in before the nurse and this* (fall prevention measures) *could be orientated by us too*” (NU1).

“*As a pharmacist myself* (...), *in the other places I’ve worked, we’ve done very little to prevent falls*” (FA2).

Although most of the focus group participants pointed out that fall prevention care could be a shared responsibility among all team members, one of the professionals emphasized that interprofessional fall prevention actions should be developed under the leadership of the doctor.

“*The doctor has to be the leader of care, he’s the one who does the least care for the hospitalized patient... But he’s the leader of the situation, he’s the hospital’s representative in front of the patient, so he has to be alongside the nursing staff, alongside the auxiliaries, alongside the carers and accept the complaints, accept what the staff who take part in this care feel, refer to and not become a person like that, opposed to the benefits that the auxiliaries would do, the nursing staff... and that really happens, that happens. As much as we... I’m corporatist towards doctors, but there are doctors who are really annoying, they always complain, they always want to do less and you have more expertise on how to deal with these situations*” (ME1).

### Health Education on Fall Prevention for Patients and Carers

Health education on fall prevention for patients and carers was not as convergent and predominant in FG1 and FG2 as the previous categories, but it was elaborated in the analysis process, given the frequent reference to the need for educational actions with patients and the consistency of this with the object of study. It is understood that health education about the risk factors for falls and preventive measures is part of specific care for fall prevention and can promote partnership between professionals, patients and carers during hospital care and during the process of transition and continuity of care after discharge. Health education has the potential to transform the patient’s/companion’s reality, since it can broaden their understanding of the risks to which they are exposed and the need for their involvement in care actions - promoting self-care.

“*I think most of the resistance to counselling is precisely the clinical condition. They* (patients) *don’t accept that two days ago they* (patients) *were at home walking alone and living alone and that here in hospital they* (patients) *need support during their hospitalization. I think that’s what most of the difficulties are, in terms of understanding what they* (patients) *are going through, the moment they’re going through... even young patients have a lot of resistance, they* (patients) *say: ‘gee, but I’m 40, I’m young, there’s nothing wrong with me, I’m not going to fall...’ I think it’s as if we* (the healthcare team) *were at a hearing, we’re not from the* (legal) *field, we’re not lawyers, so we don’t understand their language... no matter how well educated we are, how much we’ve studied at university, we don’t understand* (a reference to patients being laypeople and not understanding the healthcare team’s concerns about the risk of falling, as if any healthcare professional were at a legal hearing and didn’t understand the legal terms because they were laypeople). *It’s the same thing, I think we* (the healthcare team) *need to try to see their* (the patient’s) *side, for example the young patient in hospital, hospitalized, I think this is relevant because of* (the patient’s) *fear of understanding*” (EN1).

### Permanent Education for Falls Prevention

In both Focus Groups (FG1 and FG2), some participants referred to the need to educate professionals to prevent falls, although less frequently than the presence of health education for patients/carers. A different understanding of professional education was also observed in the two Focus Groups, since in FG1 the educational actions of health professionals were related to the perception of patients’ needs. In FG2, only the need to educate professionals about the risk and prevention of falls was mentioned.

This category was defined as the periodic and systematic education of health professionals on the protocols and guidelines for fall prevention, with the aim of learning about the concepts related to risk factors and measures for fall prevention. Although this theme was not convergent and predominant in the two Focus Groups, it was elaborated as a category given its coherence and consistency with the object of study.

“(...) *I’ve had a few patients complain when it’s time for the pantry to serve the meal, the waiters are instructed to pull out the table, leave the tray on the table, but they often don’t put the dining table near the patient. And often he* (the patient) *can’t get up, he’s unaccompanied and can’t get help to pull up the table. So, they* (patients) *say: ‘gee, the kitchen staff came round, put the meal on, but I can’t pull it up’. This is something I’ve realized over the course of my experience, so we end up advising the housekeepers to, if possible, leave the tray with the meal next to the patient... Many of them* (porters) *are afraid, sometimes they* (patients) *have some device, they have a tripod* (IV stand) *with medication and then they don’t want to run the risk of accidentally taking some medication, bumping into something... but we always advise them to try to leave the table closer so that the patient can reach it when they want to eat, as well as calling someone*...” (NU1).

“*As for the staff, I think they need to be aware of the basics, of what’s important, of what a fall can cause, of the process of hospitalizing the patient, of the very process of care that the professional is going to provide to that patient...*” (FT2).

## DISCUSSION

The results found were predominantly about the study participants’ perception and experience of fall prevention and referred to communication between healthcare professionals and patients/carers and interprofessional communication as fundamental to ensuring patient safety.

Communication between healthcare professionals and patients/carers is a key element in preventing falls and in this study communication was treated according to a dialogical concept rather than the more usual concept of message transmission^(28)^.

Interprofessional communication between the different professionals on the team involved in caring for patients and companions in hospitalization units was also dominant in the debates between the participants and appears in the literature on teamwork and interprofessional collaboration as a fundamental competence for integrating care actions to guarantee patient safety and quality healthcare^(17-26,29)^.

As for clarifying roles, the recognition of their own work and the work of other professional categories stood out. Participants referred to the actions carried out by each person in preventing falls, but rarely the actions of the other professionals in the team^(17-26,29)^.

Health education is directly related to establishing adequate communication between health professionals and patients/carers, but for this to be successful, health professionals need to be proficient in fall prevention practices, which demonstrates the importance of continuing education on the subject. It is noteworthy that permanent and continuing education was the least predominant category, but highly relevant in the context of care and fall prevention.

Although focus group 2 was made up of participants with more life and professional experience than the participants in focus group 1, there was a very favorable atmosphere at both times with each focus group, which made it possible to identify very relevant findings in the context of professional perceptions and experiences about their interactions and communication with patients and family members/carers and their effects on fall prevention practices and the relationship between teamwork and collaborative practice and fall prevention.

The number of participants was different in the two focus groups, as mentioned above, and although both groups showed convergence on the themes constructed as categories and subcategories, some themes emerged in a particular way in each focus group, as described below:

Focus group 1

**Category: Communication between professionals and patients/carers to prevent falls, (subcategories present only in this group):** Use of technology can help communication between patients and the healthcare team to prevent falls; Importance of carer participation in care to prevent falls.

**Category: Permanent education for falls prevention (subcategory present only in this group):** The need for professional education on the risk and prevention of falls and the importance of realizing what patients’ needs are.

Focus group 2

**Category: Communication between professionals and patients/carers to prevent falls, (subcategories only present in this group):** Importance of patient and carer participation in care for falls prevention; Hospital bed technologies are insufficient for communication in moments of danger between patients and healthcare professionals for falls prevention; The bond between patients and professionals is important for establishing good communication.

**Category: Interprofessional communication for falls prevention (subcategory present only in this group):** Difficulty in interprofessional communication for physical and technological reasons.

**Category: Permanent education for falls prevention (subcategory present only in this group):** Need for education on risk and prevention of falls for professionals.

In focus group 2, there were reports that referred to the difficulty of interprofessional communication for two reasons: physical distance between some professional categories and the use of different electronic systems among health professionals, making it difficult to access the records of other professionals, leading to a greater demand for time to manipulate two different systems for accessing electronic medical records.

In the studied hospital, there were two electronic medical record systems: one used by the doctor for prescriptions and progress and the other for other health professionals to record their information (assessments, vital signs measurements, etc.). If the non-medical professional wanted to access a medical professional’s information, they would have to access another system, requiring more time between opening and closing windows, as well as many clicks on the computer.

According to the participants, the time spent handling two systems had an impact on the time available for bedside care and interaction with other professionals, making it difficult to communicate with patients/carers and other health professionals.

The technology present in some medical equipment was also listed by the participants in focus group 1 as a subcategory of communication, i.e. the presence of audible and visual alarms to alert staff to unsafe patient movements was perceived as an important communication channel between patients/carers and healthcare professionals, generating greater staff attention in moments of risk and danger, positively impacting on fall prevention.

On the other hand, there were counterpoints, since the participants in focus group 2 said that technology is not enough for effective communication between health professionals and patients/carers, since risk behavior and resistance to complying with recommendations were perceived as factors that could not be overcome only by technology.

The participants in focus group 2 said that communication is the starting point for establishing good interprofessional relationships and interactions and good relationships and interactions with patients and carers.

From this, it was said, through speeches full of perceptions and experiences, that the presence of a bond between the patient/companion and the healthcare professionals can foster increased attention to the information transmitted by the healthcare team about the risk and prevention of falls and, therefore, the way of interacting, connecting and communicating with patients and carers could favor or hinder the process of health education, especially on fall prevention measures, with the aim of achieving the patients/carers understanding of the risk factors present and the importance of adopting preventive measures.

The subcategory of the need for professional education on the risk and prevention of falls and the importance of realizing what patients’ needs are emerged only in focus group 1.

The participants in focus group 1 reported that it is important to ensure the education of health professionals from different areas so that they can adopt good practices for fall prevention and ensure patient safety. In this focus group, some health professionals emphasized the need to develop the ability to perceive patients’ needs and proactively anticipate them in order to help them as needed.

While focus group 1 reported the need for professional education on the risk and prevention of falls based on recognizing patients’ needs, focus group 2 reported the need for professional education on the risk and prevention of falls, without mentioning looking at patients’ needs. The professional’s perception is that professional education is a way of raising awareness among professionals, patients and family members, but without pointing out its relationship with the identification of patients’ needs.

The results of this study corroborate national and international literature on the importance of developing competences for interprofessional collaborative practice in order to guarantee better health outcomes, especially in relation to patient safety.

Communication between professionals and patients/carers should be carried out strategically, with appropriate language for a good understanding of the subject, according to the individual needs of the patients, with a personalized approach by the health professional, which also corroborates the competences described in the Canadian reference framework on interprofessional collaboration competences^(25)^, by defining that professionals should be able to share the information needed to coordinate care with each other and with patients/family members to avoid gaps, redundancies and errors that affect both the effectiveness and efficiency of care provision.

Professionals need to develop behavioural and communication skills in order to achieve objectives related to patient safety, health education and the ability to influence, inducing the adoption of safe behaviors by patients and companions^(29)^.

The findings of this study corroborate the Global Action Plan for patient safety^(29)^, which adopts as a strategic objective the involvement and empowerment of patients and their families to help and support them on the journey of care, ensuring that it is safer. Patients, companions and carers have knowledge gained from their experiences, which is valuable in care processes, as it can be a barrier to the occurrence of adverse events.

A qualitative study carried out in Iowa^(30)^ explored the perspectives of each member of the interprofessional team in relation to the following themes: communication patterns within the interprofessional healthcare team and the influence of hospital practices and organizational elements. On the subject of communication patterns within the interprofessional team, the study showed that this occurred in a safe manner, with pertinent information about the risks between the members of the interprofessional team, but when the subject was the influence of practices, it was realized that the team understood that the responsibility for identifying the risk of falling and the adoption and prevention of falls was a practice restricted to the nursing team, without any weight of responsibility for the other professional categories, in accordance with the professional identity constructed during their training, fragmenting the responsibility for care into specific actions of a strictly disciplinary nature^(30)^.

Although the literature points to the importance of person-centred care and the professional development of competences for collaborative health practice, with a view to better results in terms of patient safety, this study contributed to the understanding that there are many challenges to the practical application of these concepts.

One of the limitations of this study was the absence of some participants in Focus Group 1 (social worker, physiotherapist and psychologist), who were unable to attend due to unforeseen demands in the care setting, a situation that limited the analysis of information produced in a group with fewer participants than planned.

This study was limited to qualitatively analyzing professionals in two focus groups and, in view of the results expressed, it is clear that further research with an approach aimed at a larger number of professionals in other Focus Groups is needed to further explore experiences and perceptions on this subject.

## CONCLUSION

This study has shown that teamwork and collaborative practice are important for achieving good results in the prevention of falls in hospital care, but for this to happen, health professionals need to acquire the necessary skills for collaborative action, especially communication with patients/carers and interprofessional communication between members of the inpatient unit’s health team. The participants’ experiences also highlight the need to recognize their own role and that of the other members of the healthcare team in preventing falls, showing a fragile knowledge of the professional role of the other areas of healthcare.

There is a need for investment in interventions in day-to-day care practices and in research aimed at developing skills that enable health professionals to become effective health educators, capable of positively influencing patients, carers and communities to adopt safer behaviors within the context of care and fall prevention, whether in hospital care or in the continuum of care.
