# Supplementary material for: Interprofessional approach to fall prevention in hospital care
Source: Rev Esc Enferm USP. 2024 Jul 5;58:e20230239. doi: 10.1590/1980-220X-REEUSP-2023-0239en (PMC11243146; doi:10.1590/1980-220X-REEUSP-2023-0239en)
Supplement: Translation

# Translation: Abordagem interprofissional na prevenção de quedas na assistência hospitalar

**DOI:** 10.1590/1980-220X-REEUSP-2023-0239pt

**Authors:** Anna Carolina da Silva Albertini, Marina Peduzzi

**Affiliations:** 1 Universidade de São Paulo, Escola de Enfermagem, São Paulo, SP, Brasil.; 2 Hospital Sírio-Libanês, São Paulo, SP, Brasil.

**Keywords:** Equipe de Assistência ao Paciente, Relações Interprofissionais, Acidentes por Quedas, Hospitais

## Abstract

**Objetivo:**

Compreender a percepção e a experiência vivenciada pelos profissionais de saúde quanto às práticas de prevenção de quedas na unidade de internação hospitalar.

**Método:**

Trata-se de um estudo qualitativo do tipo exploratório e descritivo, na modalidade estudo de caso, desenvolvido com base no referencial canadense de competências interprofissionais. Os dados foram coletados através de dois grupos focais, com profissionais de saúde diferentes em cada grupo, utilizando análise de conteúdo temática.

**Resultados:**

Foram elaboradas cinco categorias que mostraram intensa convergência entre os participantes dos dois grupos focais, dentro do contexto das práticas para prevenção de quedas: comunicação entre profissionais e pacientes/acompanhantes para a prevenção de quedas, comunicação interprofissional para a prevenção de quedas, clarificação de papéis para a prevenção de quedas, educação em saúde sobre risco e prevenção de quedas e educação permanente para a prevenção de quedas.

**Conclusão:**

O trabalho em equipe e a prática colaborativa são importantes para atingir bons resultados na prevenção de quedas na assistência hospitalar, mas para isso os profissionais de saúde precisam se apropriar das competências necessárias para o agir colaborativo.

## INTRODUÇÃO

A queda no ambiente hospitalar é um dos motivos de preocupação para os profissionais de saúde, já que aproximadamente 37,3 milhões de quedas requerem cuidados a cada ano e cerca de 684.000 pessoas morrem por este motivo^(1)^.

A ocorrência deste evento adverso no ambiente hospitalar implica em aumento da morbimortalidade, aumento do tempo de hospitalização, trazendo prejuízos físicos e psicológicos, com consequente elevação dos custos assistenciais e aumento da judicialização nos serviços de saúde^(2-4)^.

No Brasil, a queda é o terceiro evento adverso mais notificado pelo Sistema Notivisa da ANVISA (Agência Nacional de Vigilância Sanitária)^(2)^. Ocorre dano em 30% a 50% dos casos notificados, incluindo dano leve (por exemplo escoriações, hematomas), dano moderado (sangramento, entorses), dano grave (fraturas de fêmur, quadril e traumas de crânio) e dano catastrófico (perda funcional parcial ou total e óbito)^(1-4)^.

Vários são os fatores que contribuem para o aumento do número de quedas em pacientes internados: distúrbio de equilíbrio relacionados à perda de força muscular de membros inferiores e alteração de marcha, hipotensão, anemia, paresias, osteoartrites, distúrbios neurológicos, amputação, caquexia ou obesidade severa, comprometimento sensorial (visão, audição, tato), jejum, dor intensa, curativos que podem prejudicar a mobilidade do paciente e uso de dispositivos para marcha (muletas, bengalas, andadores)^(1-5)^.

Muitas são as recomendações para a prevenção de quedas, dentre as mais comuns podemos destacar a importância da capacitação dos profissionais de saúde e as orientações aos pacientes, familiares e acompanhantes sobre as boas práticas para prevenção de quedas^(6-12)^. As orientações referem-se à necessidade de manter o leito em posição baixa com as grades elevadas e travadas, a campainha sempre ao alcance para o acionamento da equipe assistencial quando necessário, itens pessoais também ao alcance do paciente e manter sinalização visual com identificação de acordo com o risco de queda do paciente (pulseira ou sinalização beira leito), visando alertar toda equipe assistencial envolvida no cuidado^(6-12)^.

A realização periódica de rondas no quarto do paciente também é uma prática estratégica para a minimização do risco de queda, uma vez que a equipe assistencial consegue constatar com antecedência as necessidades do paciente (ida ao banheiro, colocação de óculos, próteses etc.), antes que ele se levante ou movimente, especialmente se o paciente estiver desacompanhado, condição que eleva ainda mais o risco de queda^(6-12)^.

O acompanhante tem papel essencial no contexto da prevenção de quedas, já que ele é o primeiro a alertar e acionar a equipe assistencial nos momentos de maior risco e perigo para o paciente. O empoderamento do paciente e dos familiares/acompanhantes, com o envolvimento de todos no cuidado, colaboram para que os resultados sejam positivos para a prevenção de quedas, visto que esta prática está relacionada à atenção centrada no paciente e, consequentemente, com a prática interprofissional^(6-12)^.

A literatura aponta as necessidades de envolvimento de toda a equipe de saúde na prevenção de quedas na assistência hospitalar, de modo que todas as categorias profissionais envolvidas no cuidado direto aos pacientes deveriam ser bem treinadas para identificar corretamente os pacientes com moderado e alto risco de queda e serem capazes de propor e executar estratégias preventivas eficazes, com a participação ativa do paciente e do familiar/acompanhante^(6-12)^.

O presente estudo teve como objetivo analisar a percepção e experiência dos profissionais de saúde da equipe multiprofissional sobre as práticas de prevenção de queda em unidade de internação hospital.

## MÉTODO

### Tipo de Estudo

Trata-se de um estudo qualitativo do tipo exploratório e descritivo do tipo estudo de caso. Este propicia a investigação em profundidade de fenômeno no local onde ocorre, sendo utilizado na abordagem de um conjunto de eventos contemporâneos ainda pouco explorados^(13)^.

O estudo exploratório tem como objetivos analisar um tema pouco estudado, bem como os fenômenos desconhecidos ou novos, pesquisar novos problemas, identificar conceitos e estabelecer prioridades para novos estudos que serão realizados. A pesquisa descritiva busca observar, registrar e especificar as características do fenômeno que será analisado e mostrar, com precisão, as dimensões de um contexto ou situação. O pesquisador deve definir o que será observado e quais dados serão coletados^(13)^.

O estudo de caso propicia a investigação de um fenômeno em profundidade, no local onde ocorre, sendo utilizado quando se pretende investigar o como e o porquê de um conjunto de eventos contemporâneos ainda pouco explorados^(14)^.

### Local do Estudo

O estudo foi desenvolvido em um hospital localizado no município de São Paulo que atende a mais de 40 especialidades, com 512 leitos de internação, Pronto Atendimento, Centro Cirúrgico, Unidades Críticas e Centros de Medicina Avançada. O hospital conta com a acreditação pela *Joint Commission International* (JCI), desde 2007.

### Participantes do Estudo

Participaram do estudo profissionais de saúde de diversas áreas, com formação de nível técnico e de nível superior, que atendiam aos três critérios de inclusão: atuar no hospital estudado há pelo menos um ano, ter experiência de trabalho em unidade de internação e, em especial, com os cuidados de prevenção de quedas e assegurar a participação das diversas categorias profissionais.

### Procedimentos Para a Coleta de Dados

Optou-se pelo grupo focal como método de coleta de dados, visto que as interações entre os participantes poderiam contribuir para a produção e a análise das percepções e experiências dos diversos profissionais da equipe de saúde sobre os cuidados com prevenção de quedas. A pesquisadora principal do estudo assumiu a posição de coordenadora em ambos os grupos focais, facilitando as interações por meio de questões norteadoras que versaram sobre o Fluxo e Diretrizes para a Prevenção de Quedas aplicadas no hospital estudado, as experiências dos participantes quanto às ações e responsabilidades de cada categoria profissional e sobre a participação dos demais profissionais nas práticas de prevenção de quedas e as percepções e experiências sobre a participação do paciente e seus familiares/acompanhantes.

Foram realizados dois grupos focais para os quais foram convidados dois profissionais diferentes das seguintes áreas: enfermagem (enfermeira e técnicos de enfermagem), farmácia, fisioterapia, medicina, nutrição, psicologia e serviço social. No Grupo Focal 1 (GF1) participaram cinco profissionais de saúde: um farmacêutico, uma enfermeira, um médico, uma nutricionista, uma técnica de enfermagem. Não houve participação do assistente social, do fisioterapeuta e da psicóloga em razão da alta demanda na assistência e necessidade de apoio desses profissionais no cenário de Covid-19 na data quando o grupo focal havia sido agendado de comum acordo com os participantes. No Grupo Focal 2 (GF2) participaram oito profissionais de saúde: uma assistente social, uma enfermeira, uma farmacêutica, um fisioterapeuta, uma médica, uma nutricionista, uma psicóloga e um técnico de enfermagem.

Cada um dos grupos focais (1 e 2) contou também com a participação de uma observadora: ambas eram enfermeiras, com mestrado concluído em ciências da saúde, e atuavam no hospital estudado, respectivamente, há 19 anos e 14 anos.

Embora a coleta de dados tenha ocorrido durante a pandemia da Covid-19, foi possível realizar os dois grupos focais na modalidade presencial, em uma sala de reuniões com recursos de gravação de imagem e voz, após o consentimento dos participantes, por meio da assinatura do Termo de Consentimento Livre e Esclarecido. O grupo focal foi iniciado com breve apresentação dos participantes, explicação sobre o objetivo da pesquisa e apresentação do protocolo de prevenção de quedas na instituição. Os encontros aconteceram no mês de junho de 2021, cada um com duração de 02 horas.

As questões norteadoras para a condução dos grupos focais foram as seguintes:

1. Qual é sua opinião sobre o Fluxo e Diretrizes para Prevenção de Quedas aplicadas atualmente na instituição? Existe alguma prática, algum aspecto que mereça ser melhor discutido?
2. Como cada um de vocês, pensando no papel que têm em suas responsabilidades e competências profissionais, pode contribuir para a Prevenção de Queda na Unidade de Internação Hospitalar?
3. Queremos ouvir as suas experiências sobre a participação do paciente e seus familiares/acompanhantes nas práticas de prevenção de quedas.
4. Queremos ouvir as suas experiências quanto à participação dos profissionais de diversas áreas nas práticas de prevenção de quedas.
5. Na opinião de vocês, quais são os principais desafios e dificuldades para Prevenção de Quedas na unidade de internação hospitalar?
6. Para encerrar, alguém gostaria de fazer mais alguma sugestão, contribuição, comentário?

### Análise dos Dados

Para a análise dos dados gerados do grupo focal, foi empregado o método de análise de conteúdo de Bardin^(15)^, que procura conhecer aquilo que está por trás das palavras, portanto latente, buscando a aproximação com o recorte de realidade estudado por meio dos relatos e trocas entre os participantes e do contexto de cada relato, ou seja, qual a categoria profissional do participante. Foram seguidas as três etapas de análise de conteúdo temática: pré-análise, com transcrição dos dois grupos focais pela pesquisadora principal e leitura flutuante do material empírico; exploração do material, com sua codificação e, a partir desta, a construção de subcategorias e categorias com reiteradas revisões voltando a leitura do material empírico e validação com a segunda pesquisadora, orientadora do estudo; e tratamento dos resultados obtidos e interpretação, na qual foram propostas inferências e interpretações para a construção das categorias^(15,16)^.

Foi realizada a análise de cada grupo em separado e depois a análise transversal dos dois grupos. No início da análise foi utilizada a lógica dedutiva com base no referencial do modelo canadense de competências colaborativas, mas gradativamente também foi utilizada a lógica indutiva para a elaboração das subcategorias e categorias. Todo o processo de análise de conteúdo foi realizado com base no referencial teórico de trabalho em equipe e colaboração interprofissional^(17-22)^ e de competências colaborativas^(23-26)^, e também apoiado na literatura sobre prevenção de quedas.

### Aspectos Éticos

Foram respeitados os aspectos éticos e legais contidos na resolução nº466/2012 do Conselho Nacional de Saúde (CNS)^27)^, sendo o estudo submetido à avaliação do Comitê de Ética e Pesquisa (CEP) do hospital estudado, com aprovação (parecer n°4.210.393, de 2020 e parecer n°1628, de 2020). A pesquisa foi realizada após assinatura do Termo de Consentimento Livre e Esclarecido pelos participantes dos dois grupos focais. Para garantir o sigilo e a proteção dos dados dos participantes, identificamos os participantes por meio de código dos Grupos Focais 1 e 2 e da categoria profissional: assistente social (AS1 e AS2), enfermeiro (EN1 e EN2), farmacêutico (FA1 e FA2), fisioterapeuta (FT1 e FT2), médico (ME1 e ME2), nutricionista (NU1 e NU2), psicólogo (PS1 e PS2), técnico de enfermagem (TE1 e TE2).

## RESULTADOS

No Grupo Focal 1 (GF1) participaram no hospital onde foi realizada a pesquisa: cinco profissionais de saúde: uma enfermeira (EN1), 32 anos de idade, nove anos de formação e oito anos de atuação; um farmacêutico (FA1), 33 anos de idade, 12 anos de formação e dois anos de atuação; um médico (ME1), 63 anos de idade, 40 anos de formação e 38 anos de atuação; uma nutricionista (NU1), 28 anos de idade, sete anos de formação e dois anos de atuação; uma técnica de enfermagem (TE1), 42 anos de idade, nove anos de formação e sete anos de atuação.

No Grupo Focal 2 participaram oito profissionais de saúde no hospital onde foi realizada a pesquisa: uma assistente social (AS2), 35 anos de idade, 13 anos de formação e 11 anos de atuação; uma enfermeira (EN2), 30 anos de idade, oito anos de formação e sete anos de atuação; uma farmacêutica (FA2), 32 anos de idade, oito anos de formação e dois anos de atuação; um fisioterapeuta (FT2), 37 anos de idade, 13 anos de formação e 11 anos de atuação; uma médica (ME2), 41 anos de idade, 16 anos de formação e nove anos de atuação; uma nutricionista (NU2), 44 anos de idade, 18 anos de formação e dois anos de atuação; uma psicóloga (PS2), 57 anos de idade, 34 anos de formação e sete anos de atuação; um técnico de enfermagem (TE2), 54 anos de idade, 23 anos de formação e 21 anos de atuação.

Em ambos os Grupos Focais, todos os participantes demonstraram se sentir à vontade para interagir e expressar suas experiências e percepções.

A análise do material empírico do GF1 e GF2 resultou na elaboração de cinco categorias que mostraram intensa convergência entre os participantes dos dois grupos focais, embora também tenham sido analisadas divergências entre os participantes de cada grupo focal e entre os dois grupos (GF1 e GF2), como consta na Figura 1.

**Figura 1:** Este é um artigo de acesso aberto distribuído sob os termos da Licença de Atribuição Creative Commons.

### Comunicação Entre Profissionais e Pacientes/Acompanhantes Para a Prevenção de Quedas

Os participantes dos dois Grupos Focais (GF1 e GF2) mostraram que na sua percepção e experiência a comunicação entre profissionais de saúde e pacientes/acompanhantes constitui um elemento chave nas práticas de prevenção de quedas. A comunicação entre profissionais e pacientes/acompanhantes foi o tema predominante nos dois grupos focais, entendida como efetiva nas ações de prevenção se acompanhada de escuta ativa, no sentido de mostrar ao paciente interesse genuíno em ouvi-lo, de empatia e da utilização de linguagem de fácil entendimento. Os profissionais de saúde também apontaram que a comunicação com os pacientes/acompanhantes sobre os fatores de risco e as medidas preventivas de queda requer a identificação de necessidades e preferências do paciente no contexto do cuidado. Ou seja, a interação e a comunicação em torno da prevenção de quedas precisa fazer sentido para pacientes e acompanhantes no momento que ocorre.

“*Quantas vezes a gente entra e está conversando com o paciente e ele não olha pra gente* (profissional de saúde)*? Está mexendo no celular, ele não quer* (...), *ele está falando ali pra você: ‘eu não quero falar contigo’, então você* (profissional de saúde) *tem que ter a sensibilidade de voltar e falar assim “esse paciente eu volto depois*” (NU2).

“*Como técnica de enfermagem, eu acredito que a contribuição que eu conseguiria dar para o paciente seria uma escuta ativa, porque é bem diferente você ouvir do que você escutar. Ouvir refere-se a parte dos sentidos e às vezes a gente fala para o paciente: ‘Aqui está o folder, me chama quando tiver necessidade, as grades terão que ficar levantadas e o senhor pode assinar aqui’* (referindo-se ao documento de ciência sobre as medidas de prevenção de quedas). *Aí ele* (paciente) *assina, qual é a percentagem de conteúdo que esse paciente de fato conseguiu absorver disso?* (TE1).

Os participantes do GF2 referiram que na sua experiência, o estabelecimento de vínculo entre pacientes e profissionais de saúde também está relacionado à boa comunicação entre eles e ajuda a garantir a segurança do paciente, pois o vínculo facilita a maior adesão dos pacientes e acompanhantes às recomendações preventivas propostas pelos profissionais. Contudo, a relação entre vínculo e comunicação efetiva na prevenção de quedas não apareceu no GF1.

“*Então quando você* (profissional de saúde) *já tem um vínculo* (com o paciente) *e quando você já tem essa conexão, essa experiência de adesão é 10 milhões de vezes mais efetiva, eficiente* (...). *E quando interna no mesmo andar, a enfermeira diz: ah doutora, a gente* (equipe de saúde) *já conhece o paciente que está internando naquela unidade e já sabemos das suas particularidades. Então eu acho que assim, a presença de um vínculo prévio à internação, eu acho que melhora a adesão e a experiência* (do paciente) *nas nossas práticas de cuidado como um todo, entre elas a prevenção de quedas*” (ME2).

A maioria dos participantes referiu a comunicação com pacientes e acompanhantes como um componente que contribui para a segurança do paciente. Contudo, um dos profissionais fez referência apenas ao envolvimento do acompanhante no cuidado para com a segurança do paciente, remetendo a necessidade do seu entendimento sobre os fatores de risco de queda.

“*O acompanhante está o maior tempo no quarto, aí quando é feito algum medicamento e tem alguma reação adversa, agitando o paciente e ele* (acompanhante) *não compartilha* (com a equipe), *mas é importante ele ter a informação* (efeitos medicamentosos) *e compartilhar o que acontecer no quarto para prevenir quedas*” (FA1).

Foi observada divergência entre os grupos GF1 e GF2 quanto à interferência da tecnologia nos cuidados da prevenção de quedas, visto que no GF1 foi apontada a importância da tecnologia, mas no GF2 foi questionado o quanto a tecnologia pode ajudar a prevenir as quedas com os alarmes no leito.

“*E acredito que da prevenção, diretrizes de prevenção* (de quedas) *que a gente* (equipe de saúde) *utiliza, melhorou bastante em* relação, por exemplo, a instituição de camas com alarmes no hospital (camas com alarme de movimento que avisam a equipe quando o paciente tenta se levantar sozinho, sem pedir ajuda), *principalmente para pacientes que estão em unidades críticas, que acontece mesmo deles* (pacientes) *levantarem, tentarem levantar e isso ajudou bastante a gente* (equipe de saúde), *de alarmar a cama e a gente* (equipe de saúde) *entrar no quarto*” (EN1).

“*As camas do hospital, eu acho de última geração, mas no meu entender elas não são funcionais para o risco de queda, porque você* (profissional de saúde) *entra no quarto, você orienta, você levanta as grades* (do leito), *aí quando você chega, o paciente está no banheiro! As grades* (do leito) *estão elevadas, por onde ele* (paciente) *saiu? Pela beirada* (da cama)*... Mesmo com o alarme acionado... Porque tem um espaço grande onde ele passa* (espaço sem grade no rodapé da cama). *Então, muitas vezes o paciente sai, volta pra cama e você* (profissional de saúde) *nem sabe que o paciente saiu*” (TE2).

### Comunicação Interprofissional Para a Prevenção de Quedas

Nos dois grupos focais foi apresentada a percepção e experiência dos profissionais de saúde sobre a necessidade de comunicação interprofissional para o melhor desempenho das ações relacionadas ao cuidado na prevenção de quedas.

“*A troca de informações é muito importante, passar o que ocorreu no plantão passado, o paciente ele não é um algo fixo* (...). *Ele é volátil durante o dia, ele muda muito conforme a medicação que o médico deu, que é necessária muitas vezes* (...), *então tem uma variação de força muscular, de nível de consciência* (...). *De manhã ele estava de um jeito, a tarde ele está de outro* (...), *essa troca de informações entre plantões é muito importante*” (FT2).

Um participante do GF1 mencionou uma experiência de aprendizagem no trabalho que decorreu da comunicação interprofissional com troca de saberes e colaboração, as quais possibilitaram melhor desempenho nas ações relacionadas ao cuidado para prevenção de quedas.

“*Eu tenho uma experiência bacana de trabalho com os fisioterapeutas* (...), *eu acho excepcional porque é uma conexão que a gente* (técnicos de enfermagem e fisioterapeutas) *tem* (...). *Eu estou lá fazendo algum procedimento, o fisioterapeuta entra* (...). *Eu estou trocando a fralda, e o fisioterapeuta me diz: “Você quer dar um banho agora? Eu já vou te auxiliar porque esse paciente, pelo o que eu já trabalhei ontem, sei que ele tem uma dificuldade aqui ou ali. Às vezes eu* (técnica de enfermagem) *já recebi o plantão do paciente, já sei quais são as dificuldades dele, mas ainda não senti de fato como será essa dificuldade, então eu acho que é muito bom ter a sinergia que a gente tem lá*” (TE1).

Os participantes do Grupo Focal 2 mencionaram que há dificuldade na comunicação interprofissional por motivos físicos e tecnológicos, relacionando esta dificuldade com o uso de dois sistemas no prontuário eletrônico, sendo que um é utilizado pela equipe médica e o outro é utilizado pelas demais categorias profissionais, o que dificulta o acesso às informações registradas pelo tempo gasto para o uso dos dois sistemas, o que diminui a disponibilidade do profissional à beira leito e a possibilidade de comunicação e interação com pacientes/acompanhantes e demais profissionais de saúde.

“*Eu não tenho acesso ao que a farmacêutica escreve, ao que a nutricionista escreve, ao que o fisioterapeuta escreve, ao que o técnico de enfermagem escreve, ao que a psicóloga escreve... eu tenho que acessar o sistema* (sistema de prontuário eletrônico) *e mesmo assim, deveria ser algo automático. Eu posso acessar o que vocês estão escrevendo, deveria ser muito mais fácil, porque, sem sombra de dúvida a minha ação depende muito da informação que eu tenho lá, que eu vejo. O que a gente sempre ouve é ‘mas é só acessar o sistema”, “o sistema é lento, o sistema demora, o sistema não sei o quê...” Aí o segundo sistema* (outro sistema eletrônico para realizar prescrições e visualizar o prontuário do paciente) *demora 01 hora pra liberar a prescrição, e aí você passou 15 minutos só na teoria e na prática mesmo, lá na troca que você poderia estar gastando esses 15 minutos incentivando a autonomia do paciente e estimulando a família, você tá tentando acertar o sistema pra saber o que a farmacêutica ou o que a nutricionista achou, porque você não consegue...*” (ME2).

### Clarificação de Papéis Para a Prevenção de Quedas

A clarificação de papéis também foi convergente nos dois Grupos Focais como um elemento que contribui na prevenção de quedas e expressa o entendimento que os profissionais de saúde têm sobre a necessidade de reconhecimento das ações e saberes da própria prática profissional, bem como das ações e saberes das demais áreas que compõem a equipe e que estão envolvidas nos cuidados de prevenção de quedas. O processo de clarificação de papéis da própria área profissional e das demais que integram a equipe remete à possibilidade de corresponsabilidade interprofissional nas ações de prevenção de quedas, o que constitui uma das características do trabalho em equipe integrado com colaboração interprofissional^(23-26)^.

Entretanto, os resultados mostram que os profissionais de saúde que participaram do estudo têm conhecimento, em especial, de sua contribuição específica desde a sua profissão em saúde e menos as ações e responsabilidades dos demais integrantes da equipe.

“*Como farmacêutico, a experiência que eu tive... acho que tive mais experiência agora porque eu faço parte do time de auditoria* (auditoria interna relacionada ao bundle (pacote) de prevenção de quedas) *e quando a gente entra para fazer essa auditoria eles veem que a gente como farmacêutico também está se importando, por alguma orientação que a equipe de enfermagem fez... que outros profissionais estão de olho também no risco de queda e quando a gente faz entrevista farmacêutica, a gente também orienta sobre as reações adversas dos medicamentos e muitas vezes eles não sabem que estão utilizando o medicamento que tem risco para queda*” (FA1).

Os participantes dos dois Grupos Focais referiram que a prevenção de quedas poderia ser responsabilidade de todos os membros da equipe de saúde. Porém, também ficou evidente nos GF1 e GF2 que na percepção dos participantes, as práticas realizadas no ambiente hospitalar para garantir a prevenção de quedas estão, sobretudo, sob a responsabilidade da equipe de enfermagem.

“*Eu vejo que acaba ficando muita coisa nas mãos do enfermeiro* (...), *muita coisa para orientar* (...) *eles ficam com uma responsabilidade grande nessa parte de cuidado integral e às vezes isso poderia ser multiplicado, dividido entre os outros profissionais de saúde. Realmente toda a equipe, poderia orientar sobre prevenção de queda, afinal é um cuidado com o paciente. Por exemplo, a gente tem até 24h pra fazer a nossa triagem e aí muitas vezes a gente acaba entrando antes do enfermeiro e isso* (medidas de prevenção de queda) *poderia sim ser orientado por nós também*” (NU1).

“*Eu como farmacêutica também* (...), *nos outros lugares que eu já trabalhei, a nossa atuação acaba sendo bem pequena na prevenção de quedas*” (FA2).

Embora a maioria dos participantes dos grupos focais tenham apontado que os cuidados para a prevenção de quedas poderia ser uma responsabilidade compartilhada entre todos os membros da equipe, um dos profissionais destacou que as ações interprofissionais para a prevenção de quedas seriam desenvolvidas sob a liderança do médico.

“*O médico tem que ter a posição de líder dos cuidados, é o que menos faz cuidados no paciente internado... Mas ele é o líder da situação, ele é o representante do hospital diante do paciente, então ele tem que estar ao lado da enfermagem, ao lado dos auxiliares, ao lado dos cuidadores e aceitar as queixas, aceitar aquilo que o pessoal que participa desses cuidados sente, referem e não se tornar uma pessoa assim, contrária as benfeitorias que o pessoal dos auxiliares fariam, a enfermagem... e isso acontece mesmo, isso acontece. Por mais que a gente... que eu seja corporativista em relação aos médicos, mas tem médico que é chato mesmo, eles sempre reclamam, eles sempre querem fazer menos e vocês têm mais a expertise de como lidar com estas situações*” (ME1).

### Educação em Saúde Sobre Prevenção de Quedas Para Pacientes e Acompanhantes

A educação em saúde sobre prevenção de quedas para pacientes e acompanhantes não foi convergente e predominante nos GF1 e GF2 tal como as categorias anteriores. Contudo, ela foi elaborada no processo de análise, visto a frequente referência à necessidade de ações educativas junto aos pacientes e a consistência disso ao objeto de estudo. Entende-se que a educação em saúde sobre os fatores de risco de queda e as medidas preventivas integra os cuidados específicos para prevenção de quedas e pode promover a parceria entre profissionais, pacientes e acompanhantes no decorrer do cuidado hospitalar e durante o processo de transição e continuidade do cuidado após a alta. A educação em saúde tem o potencial de transformar a realidade do paciente/acompanhante, uma vez que pode ampliar seu entendimento sobre os riscos a que está exposto e a necessidade de seu envolvimento nas ações de cuidado - promovendo o autocuidado.

“*Eu acho que a maioria das resistências em relação a orientação é justamente o quadro clínico. Eles* (pacientes) *não aceitam que há dois dias eles* (pacientes) estavam em casa andando sozinho e morando sozinho e que aqui no hospital eles (pacientes) *precisam de um amparo, durante a internação. Eu acredito que a maioria das dificuldades é essa, em relação a entender o que ele* (paciente) *está passando, o momento que está passando... mesmo o paciente jovem tem bastante resistência, eles* (pacientes) *dizem: ‘nossa, mas eu tenho 40 anos, sou jovem, não tenho nada demais, eu não vou cair...’ Eu acredito que é como se a gente* (equipe de saúde) *estivesse em alguma audiência, não somos da área* (jurídica), *não somos advogados, então não entendemos a língua deles... por mais que sejamos instruídos, tenhamos feito faculdade e estudado, nós não entendemos* (referência para falar sobre os pacientes serem leigos e não entenderem as preocupações da equipe de saúde sobre o risco de queda, bem como se qualquer profissional de saúde estivesse em alguma audiência jurídica e não compreendesse os termos legais por serem leigos no assunto). É a mesma coisa, acho que a gente (equipe de saúde) *precisa tentar ver o lado deles* (pacientes), *por exemplo o paciente jovem no hospital, internado, acredito que isso seja relevante pelo medo* (do paciente) *de entender*” (EN1).

### Educação Permanente Para a Prevenção de Quedas

Em ambos os Grupos Focais (GF1 e GF2), alguns participantes fizeram referência à necessidade de educação dos profissionais para prevenção de quedas, embora com menor frequência que a presença da educação em saúde de pacientes/acompanhantes. Também foi observado o entendimento distinto de educação dos profissionais nos dois Grupos Focais, visto que no GF1 as ações educativas dos profissionais de saúde estiveram relacionadas à percepção das necessidades dos pacientes. No GF2 foi referida apenas a necessidade de educação dos profissionais sobre risco e prevenção de queda.

Esta categoria foi definida como a ação educativa, de forma periódica e sistemática, dos profissionais de saúde sobre os protocolos e diretrizes para prevenção de quedas, visando a aprendizagem sobre os conceitos relacionados aos fatores de risco e às medidas para prevenção de quedas. Embora este tema não tenha sido convergente e predominante nos dois Grupos Focais, foi elaborada como categoria dada sua coerência e consistência com o objeto de estudo.

“(...) *Eu já tive alguns pacientes que se queixam na hora da copa servir a refeição, as copeiras são orientadas a puxar a mesa, deixar a bandeja em cima da mesa, mas muitas vezes elas não colocam a mesa de refeição próximo ao paciente. E muitas vezes ele* (paciente) *não consegue levantar, está sem acompanhante e não consegue ajuda para puxar a mesa. Então eles* (pacientes) *dizem: ‘nossa, a equipe de copa veio aqui, colocou a refeição, mas eu não consigo puxar’. Isso é algo que percebi ao longo da minha experiência, então a gente acaba orientando as copeiras de, se possível, deixar próximo ao paciente* (a bandeja com a refeição)*... Muitas delas* (copeiras) *têm receio, às vezes eles* (pacientes) *têm algum dispositivo, fica com tripé* (suporte de soro) *com medicação e aí elas não querem correr o risco de tirar alguma medicação sem querer, esbarrar em alguma coisa... mas a gente sempre orienta pra elas tentarem deixar a mesa mais perto para o paciente conseguir alcançar quando ele quiser comer, além de chamar alguém*...” (NU1).

“*Quanto à equipe, eu acho que a conscientização do básico, do que é importante, do que o que a queda pode causar, do processo de internação do paciente, do próprio processo de cuidado que o profissional vai fornecer àquele paciente*...” (FT2).

## DISCUSSÃO

Os resultados encontrados foram predominantes sobre a percepção e experiência dos participantes do estudo sobre prevenção de quedas e se referiram à comunicação entre profissionais de saúde e pacientes/acompanhantes e a comunicação interprofissional como fundamentais para a garantia da segurança do paciente.

A comunicação entre profissionais de saúde e pacientes/acompanhantes constitui elemento chave na prevenção de quedas e neste estudo a comunicação foi tratada segundo a concepção dialógica e não na concepção mais usual de transmissão de mensagem^(28)^.

A comunicação interprofissional entre os diferentes profissionais da equipe envolvida no cuidado aos pacientes e acompanhantes nas unidades de internação, também foi dominante nos debates entre os participantes e aparece na literatura sobre trabalho em equipe e colaboração interprofissional como competência fundante da integração das ações de cuidado para garantir a segurança do paciente e qualidade da atenção à saúde^(17-26,29)^.

Quanto à clarificação dos papéis destacou-se o reconhecimento do próprio trabalho e do trabalho das outras categorias profissionais, os participantes fizeram referência às ações realizadas por cada um na prevenção de quedas, mas raramente as ações dos demais profissionais da equipe^(17-26,29)^.

A educação em saúde está diretamente relacionada ao estabelecimento de uma adequada comunicação entre profissionais de saúde e pacientes/acompanhantes, porém, para que haja sucesso é necessário que os profissionais de saúde tenham proficiência sobre as práticas para a prevenção de quedas, o que demonstra a importância da educação permanente sobre o tema. Chama a atenção que a educação permanente e continuada tenha sido a categoria menos predominante, embora seja altamente relevante no contexto do cuidado e da prevenção de quedas.

Embora o grupo focal 2 tenha sido composto por participantes com maior experiência de vida e de profissão em relação aos participantes do grupo focal 1, houve um clima muito favorável nos dois momentos com cada grupo focal, que possibilitou identificar achados muito relevantes no contexto das percepções e experiências profissionais sobre as suas interações e comunicação com os pacientes e familiares/acompanhantes e os seus efeitos nas práticas de prevenção de quedas e sobre as relações entre o trabalho em equipe e prática colaborativa e a prevenção de quedas.

O número de participantes foi diferente nos dois grupos focais, como mencionado anteriormente e, embora ambos grupos tenham demonstrado convergência aos temas construídos como categorias e subcategorias, algumas temáticas emergiram de forma particular em cada grupo focal, como descrito a seguir:

Grupo focal 1

**Categoria: Comunicação entre profissionais e pacientes/acompanhantes para a prevenção de quedas, (subcategorias presentes apenas neste grupo):** Utilização da tecnologia pode ajudar na comunicação entre pacientes e a equipe de saúde para a prevenção de quedas; Importância da participação do acompanhante no cuidado para a prevenção de quedas.

**Categoria: Educação Permanente para a prevenção de quedas, (subcategoria presente apenas neste grupo):** Necessidade de educação profissional sobre risco e prevenção de quedas e a importância de perceber quais são as necessidades dos pacientes.

Grupo focal 2

**Categoria: Comunicação entre profissionais e pacientes/acompanhantes para a prevenção de quedas, (subcategorias presentes apenas neste grupo):** Importância da participação do paciente e do acompanhante no cuidado para a prevenção de quedas; As tecnologias das camas hospitalares são insuficientes para a comunicação nos momentos de perigo entre os pacientes e os profissionais de saúde para a prevenção de quedas; O vínculo entre pacientes e profissionais é importante para estabelecer uma boa comunicação.

**Categoria: Comunicação interprofissional para a prevenção de quedas (subcategoria presente apenas neste grupo):** Dificuldade na comunicação interprofissional por motivos físicos e tecnológicos.

**Categoria: Educação Permanente para a prevenção de quedas, (subcategoria presente apenas neste grupo):** Necessidade de educação sobre risco e prevenção de quedas para os profissionais.

No grupo focal 2, houve relatos que remeteram à dificuldade de comunicação interprofissional por dois motivos: distância física entre algumas categorias profissionais e uso de sistemas eletrônicos distintos entre os profissionais de saúde, dificultando o acesso aos registros de outros profissionais, levando a maior demanda de tempo para manipulação de dois sistemas diferentes para acesso de prontuário eletrônico.

No hospital estudado havia dois sistemas de prontuário eletrônico: um utilizado pelos médicos para realizar prescrição e evolução e outro para os demais profissionais de saúde registrarem suas informações (avaliações, aferições de sinais vitais, entre outros). Se o profissional não médico quisesse acessar as informações de um profissional médico, deveria acessar outro sistema, demandando maior tempo entre aberturas e fechamentos de janelas, além de muitos cliques no computador.

Segundo o relato dos participantes, o tempo gasto com o manuseio de dois sistemas repercutia no tempo disponível para atenção à beira do leito e interação com outros profissionais, dificultando a comunicação com os pacientes/acompanhantes e outros profissionais de saúde.

A tecnologia presente em alguns equipamentos médicos também foi relacionada pelos participantes do grupo focal 1 como uma subcategoria da comunicação, ou seja, a presença de alarmes sonoros e visuais para alertar a equipe sobre a movimentação insegura dos pacientes foi percebida como um importante canal de comunicação entre pacientes/acompanhantes e profissionais de saúde, gerando maior atenção da equipe em momentos de risco e perigo, impactando positivamente para a prevenção de quedas.

Por outro lado, houve contrapontos, visto que os participantes do grupo focal 2 referiram que a tecnologia não é suficiente para uma comunicação efetiva entre profissionais de saúde e pacientes/acompanhantes, visto que o comportamento de risco e a resistência em cumprir as recomendações foram fatores percebidos como não contornáveis pelas vias da tecnologia.

Por meio das falas dos participantes do grupo focal 2, percebeu-se que a comunicação é o ponto de partida para estabelecer boas relações interprofissionais e boas interações com pacientes e acompanhantes.

A partir disso, foi dito por meio de falas carregadas por percepções e experiências, que a presença de vínculo entre o paciente/acompanhante e os profissionais de saúde pode favorecer o aumento da atenção às informações transmitidas pela equipe de saúde sobre risco e prevenção de quedas e, portanto, a forma de interagir, se conectar e se comunicar com os pacientes e acompanhantes poderia favorecer ou dificultar o processo de educação em saúde, especialmente sobre as medidas de prevenção de quedas, com a preocupação de atingir a compreensão do paciente/acompanhante sobre os fatores de risco presentes e a importância da adoção de medidas preventivas.

A subcategoria necessidade de educação profissional sobre risco e prevenção de quedas e a importância de perceber quais são as necessidades dos pacientes emergiu apenas no grupo focal 1.

Os participantes do grupo focal 1 relataram ser importante garantir a educação dos profissionais de saúde de diversas áreas de atuação para que possam adotar as boas práticas para a prevenção de quedas e garantir a segurança do paciente. Neste grupo focal foi destacado por alguns profissionais de saúde que é preciso desenvolver a habilidade de percepção sobre as necessidades dos pacientes, se antecipando de forma proativa para ajudá-los no que for preciso.

Enquanto no grupo focal 1 houve relatos sobre a necessidade de educação profissional sobre o risco e prevenção de quedas com base no reconhecimento das necessidades dos pacientes, no grupo focal 2 houve um relato relacionando a necessidade de educação profissional sobre risco e prevenção de quedas, sem mencionar o olhar para as necessidades dos pacientes. A percepção do profissional é que a educação profissional é um caminho para a conscientização de profissionais, pacientes e familiares, porém sem apontar sua relação com a identificação das necessidades dos pacientes.

Os resultados deste estudo corroboram com a literatura nacional e internacional sobre a importância do desenvolvimento de competências para a prática colaborativa interprofissional, no intuito de garantir melhores resultados em saúde, especialmente em relação à segurança do paciente.

A comunicação entre profissionais e pacientes/acompanhantes deveria ser realizada de forma estratégica, com linguagem apropriada para o bom entendimento do assunto, de acordo com as necessidades individuais dos pacientes, com abordagem personalizada pelo profissional de saúde, o que também corrobora com as competências descritas no marco de referência canadense sobre as competências de colaboração interprofissional^(25)^, ao definir que os profissionais devem ser capazes de compartilhar as informações necessárias para coordenar os cuidados uns com os outros e com os pacientes/familiares para evitar lacunas, redundâncias e erros que afetam tanto a eficácia quanto à eficiência da prestação de cuidados.

Os profissionais precisam desenvolver habilidades comportamentais e comunicacionais para atingir os objetivos relacionados à segurança do paciente, da educação em saúde e da capacidade de influência, induzindo a adoção de comportamentos seguros por parte dos pacientes e acompanhantes^(29)^.

Os achados deste estudo corroboram com o Plano de Ação Global, pela segurança do paciente^(29)^, que adota como objetivo estratégico o envolvimento e a capacitação de pacientes e seus familiares para ajudar e apoiar na jornada do cuidado, garantindo que ele seja mais seguro. Os pacientes, acompanhantes e cuidadores possuem conhecimentos adquiridos em suas experiências, que são valiosas nos processos do cuidado, visto que podem ser barreiras para a ocorrência de eventos adversos.

Um estudo qualitativo, realizado em Iowa^(30)^, explorou as perspectivas de cada membro da equipe interprofissional em relação aos temas: padrões de comunicação na equipe interprofissional de saúde e influências das práticas e elementos organizacionais do hospital. Sobre o tema padrão de comunicação da equipe interprofissional, o estudo apontou que a comunicação ocorria de maneira segura, com as informações pertinentes sobre os riscos entre os membros da equipe interprofissional, mas quando o tema era a influência das práticas, percebeu-se que a equipe entendia que a responsabilidade de identificação do risco de cair e a adoção e prevenção de quedas era uma prática restrita à equipe de enfermagem, sem qualquer peso de responsabilidade para as outras categorias profissionais, de acordo com a identidade profissional construída durante sua formação, fragmentando a responsabilidade assistencial para ações específicas e de cunho estritamente disciplinar^(30)^.

Embora a literatura aponte para a importância do cuidado centrado na pessoa e no desenvolvimento profissional das competências para a prática colaborativa em saúde, visando melhores resultados no que se refere à segurança do paciente, o presente estudo contribuiu para a compreensão de que existem muitos desafios para a aplicação prática destes conceitos.

Uma das limitações deste estudo foi a ausência de alguns participantes no Grupo Focal 1 (assistente social, fisioterapeuta e psicólogo), que não puderam comparecer pelas demandas imprevistas no cenário assistencial, situação que limitou a análise de informações produzidas em um grupo com menor número de participantes do que o planejado.

Este estudo se limitou a analisar qualitativamente profissionais em dois grupos focais e, diante dos resultados expressados, fica evidente a relevância de aprofundar as pesquisas com abordagem direcionada a um maior número de profissionais em outros Grupos Focais, para explorar ainda mais as experiências e percepções sobre esta temática.

## CONCLUSÃO

O presente estudo evidenciou que o trabalho em equipe e a prática colaborativa são importantes para atingir bons resultados na prevenção de quedas na assistência hospitalar, mas para isso os profissionais de saúde precisam se apropriar das competências necessárias para o agir colaborativo, com destaque para comunicação com os pacientes/acompanhantes e comunicação interprofissional entre os membros da equipe de saúde da unidade de internação. As experiências dos participantes também destacam as necessidades de reconhecimento do próprio papel e dos demais integrantes da equipe de saúde para a prevenção de quedas, evidenciando frágil conhecimento sobre o papel profissional das demais áreas da saúde.

É necessário investimento em intervenções nas práticas do dia a dia assistencial e em pesquisas voltadas para o desenvolvimento de capacidades que permitam que os profissionais de saúde se tornem efetivos educadores em saúde, capazes de influenciar de forma positiva os pacientes, acompanhantes e comunidades a terem comportamentos mais seguros, dentro do contexto do cuidado e da prevenção de quedas, seja na assistência hospitalar ou na continuidade do cuidado.
